# Artificial intelligence–assisted breast ultrasound: modest AUROC improvement and shorter interpretation time without significant change in diagnostic accuracy

**DOI:** 10.3389/fradi.2026.1747783

**Published:** 2026-03-18

**Authors:** Hyuksool Kwon, Sun Mi Kim, Seok Hwan Oh, Mijung Jang, Myeong-Gee Kim, Hyeon-Min Bae, Sang Il Choi, Su Min Cho, Youngmin Kim, Guil Jung, Hyeon-Jik Lee, Sang-Yun Kim

**Affiliations:** 1 Laboratory of Quantitative Ultrasound Imaging, Seoul National University Bundang Hospital, Seongnam, Republic of Korea; 2 Imaging Division, Department of Emergency Medicine, Seoul National University Bundang Hospital, Seongnam, Republic of Korea; 3 Barreleye Inc., Seoul, Republic of Korea; 4 Department of Radiology, Seoul National University Bundang Hospital, Seoul National University College of Medicine, Seongnam, Republic of Korea; 5 Electrical Engineering Department, Korea Advanced Institute of Science and Technology, Daejeon, Republic of Korea

**Keywords:** artificial intelligence, breast cancer, computer-aided diagnosis, diagnostic accuracy, ultrasound imaging

## Abstract

**Objectives:**

To evaluate whether Vis-BUS, a commercial artificial intelligence (AI) breast ultrasound detection and analysis software, improves diagnostic discrimination and interpretation efficiency in breast ultrasound examinations.

**Materials and methods:**

This retrospective multi-reader study included 258 breast ultrasound examinations (129 malignant and 129 benign lesions). Six radiologists independently interpreted all cases without AI and, after a two-week washout, with AI assistance. Diagnostic performance metrics, including the area under the receiver operating characteristic curve (AUROC), area under the precision–recall curve (AUPRC), accuracy, sensitivity, and specificity, were compared using multi-reader analysis. Median interpretation time per case was recorded and compared using paired statistical tests.

**Results:**

Vis-BUS assistance modestly increased the pooled AUROC (0.921 vs. 0.953, *p* = 0.002) and reduced median reading time (6.0 vs. 3.0 s, *p* < 0.001), whereas AUPRC, accuracy, sensitivity, and specificity did not differ significantly (all *p* > 0.06). Accuracy (79.1% vs. 83.9%, *p* = 0.061), sensitivity (94.2% vs. 96.3%, *p* = 0.243), and specificity (64.0% vs. 71.6%, *p* = 0.069) showed no significant differences. Median interpretation time decreased from 6.0 to 3.0 s (*p* < 0.001). Subgroup analyses demonstrated significant AUROC improvements for dense breasts and tumors ≤ 2 cm (*p* < 0.001 for both).

**Conclusion:**

Vis-BUS AI assistance was associated with improved diagnostic discrimination and shorter interpretation time. However, accuracy, sensitivity, and specificity did not differ significantly. These findings suggest potential efficiency benefits, while the clinical impact remains to be confirmed in prospective multi-center studies.

## Introduction

Breast cancer remains the most common malignancy among women worldwide and represents a leading cause of cancer-related mortality (1). Mammography is the standard screening modality; however, its diagnostic performance is significantly reduced in women with dense breast tissue, in whom cancers may be masked by fibroglandular structures (2, 3). Supplemental breast ultrasound (US) provides real-time, radiation-free imaging and demonstrates higher sensitivity for small and mammographically occult cancers (4). This clinical relevance is particularly pronounced in East Asia. In South Korea, where over 50% of screening-age women have heterogeneously or extremely dense breasts, the Korean National Health Insurance Service began reimbursing supplemental breast ultrasound in 2021 (5). The ongoing MUST-BE trial is evaluating combined mammography-plus-ultrasound screening in women aged 40–59 (6). In this context, optimizing ultrasound interpretation addresses a clinical need of national importance. Nonetheless, US interpretation suffers from operator dependence, variable specificity, and longer interpretation times, leading to higher recall rates and unnecessary biopsies compared with mammography (7).

To address these challenges, artificial intelligence (AI) solutions for breast US have been developed. AI-based computer-aided detection (CADe) and diagnosis (CADx) systems can automatically identify suspicious regions (8, 9). Prior reader studies have suggested that such AI systems may improve diagnostic accuracy, reduce inter-reader variability, and enhance efficiency (10, 11). More recently, commercial AI breast US tools have demonstrated potential to support radiologists in both diagnostic and screening settings (12). Although much of the literature comprises retrospective, laboratory-style reader studies, to our knowledge no prior study has simultaneously quantified both diagnostic discrimination and interpretation efficiency in a fully crossed multi-reader multi-case design using a commercial AI system integrated into the reading workflow with automated time logging and calibration analysis (13).

We had developed an AI breast ultrasound detection and analysis software (Vis-BUS) and this is designed to assist radiologists by providing lesion localization and analysis. Given the limitations of traditional US interpretation and the emerging role of AI, we conducted a multi-reader study to evaluate whether Vis-BUS assistance could improve diagnostic discrimination and reduce interpretation time in breast US. We hypothesized that AI support would enhance lesion-level diagnostic performance and efficiency, particularly in challenging subgroups such as dense breasts and small tumors.

## Materials and methods

### Study design and ethical approval

This was a single-center, retrospective, multi-reader, multi-case study approved by the Institutional Review Board of Seoul National University Bundang Hospital (protocol no. E-2310-858-301). The requirement for informed consent was waived because all data were de-identified and the study posed minimal risk.

### Study population

From the institutional PACS archives, 258 breast ultrasound examinations were retrospectively collected between 2015 and 2020. All examinations were diagnostic breast ultrasound studies performed for evaluation of known or suspected breast lesions; screening ultrasound examinations were not included in this cohort. Therefore, recall from screening does not apply to this study population. Eligible patients were women aged 19–79 years who underwent diagnostic breast US for solid breast lesions ≥ 5 mm with a definitive reference diagnosis. A total of 129 malignant lesions (112 invasive carcinomas and 17 ductal carcinoma *in situ* confirmed by pathology) and 129 benign lesions (55 biopsy-proven and 74 confirmed stable after ≥ 12 months of imaging follow-up) were included. The proportion of DCIS among malignant cases (17/129, 13.2%) is consistent with reported institutional and registry data, in which DCIS typically represents 10%–20% of pathologically confirmed breast cancers.

In cases with multiple lesions, only the index lesion was selected for analysis, defined as the lesion with pathologic confirmation or, if unavailable, the most clinically relevant lesion with long-term follow-up. This approach minimized redundancy and ensured independence of cases. Breast density was abstracted from the closest mammogram within ±12 months when available; density was unknown in 35/258 (13.6%) because some patients underwent diagnostic US without mammography in routine practice. We retained such cases to reflect real-world workflows but explicitly acknowledge this as a limitation and provide subgroup analyses by density where available. A prior history of breast cancer or lesion was permitted if the patient was not undergoing active cancer treatment at the time of imaging and the ultrasound was not obtained within one month after biopsy or surgery, in line with our exclusion criteria. The detailed inclusion and exclusion criteria are provided in Supplementary Material S1 and Supplementary Table S1.

### Case selection and sampling

We used an enrichment strategy to stabilize multi-reader variance: all eligible malignant lesions during the accrual window were enumerated, and benign cases were randomly sampled from the pool of eligible benign examinations to achieve a 1:1 ratio (129:129). This design increases the precision of reader-study estimates but limits the generalizability of prevalence-dependent metrics (e.g., PPV/NPV and AUPRC), which are therefore interpreted cautiously and contextualized in the Discussion/Limitations.

The accrual window (2015–2020) was chosen to ensure temporal separation from the Vis-BUS training data (acquired through 2019). All eligible malignant diagnostic breast US cases meeting the prespecified inclusion and exclusion criteria were enumerated; the resulting count of 129 reflected the application of strict eligibility requirements (solid lesions ≥5 mm, definitive reference standard, no post-procedural imaging within one month). Readers were informed that the study dataset was enriched with approximately equal proportions of malignant and benign cases, consistent with published recommendations for MRMC reader studies (14). This transparency minimizes the risk of threshold distortion associated with undisclosed enrichment.

### Image acquisition

All examinations were performed using high-resolution ultrasound systems from multiple vendors (Samsung Medison, GE Healthcare, Philips Healthcare), equipped with high-frequency linear transducers. At least two orthogonal B-mode images of each lesion were acquired and stored.

### Reader panel and reading sessions

Six board-certified radiologists independently interpreted all cases. The panel included two breast imaging specialists (14 and 17 years of experience) and four general radiologists (1–38 years of experience). Reader labeling was as follows: R1 (17 y) and R3 (14 y) were breast imaging specialists; R2 (1 y), R4 (38 y), R5 (1 y), and R6 (1 y) were general radiologists.

In session 1 (unaided), radiologists reviewed images without AI assistance. After a two-week washout, the same set of cases was re-randomized and interpreted again with AI assistance (session 2). Although readers may have recognized previously seen lesions, the washout interval and randomization of case order were intended to minimize recall bias. For each case, radiologists determined recall vs. no recall, drew a region of interest (ROI), and assigned a malignancy score.

### AI tool

Vis-BUS (Barreleye Inc., Seoul, South Korea) is a commercially available AI breast ultrasound detection and analysis software that has received regulatory clearance from the Korean Ministry of Food and Drug Safety (MFDS). The system was developed by Barreleye Inc., whose relationship to study authors is disclosed in the Conflict of Interest statement. Vis-BUS was trained on 190,000 ultrasound images obtained from five tertiary hospitals between 2010 and 2019. Expert radiologists annotated lesion boundaries and malignancy, with each annotation independently verified by a second reader. The dataset was split into 70% training, 15% validation and 15% internal test sets at the patient level to prevent data leakage. Key hyperparameters were tuned on the validation set (learning rate=1 × 10^−4^, weight decay=1 × 10^−5^, batch size=16). Additional details are provided in Supplementary Material S2.

**Figure 1:**
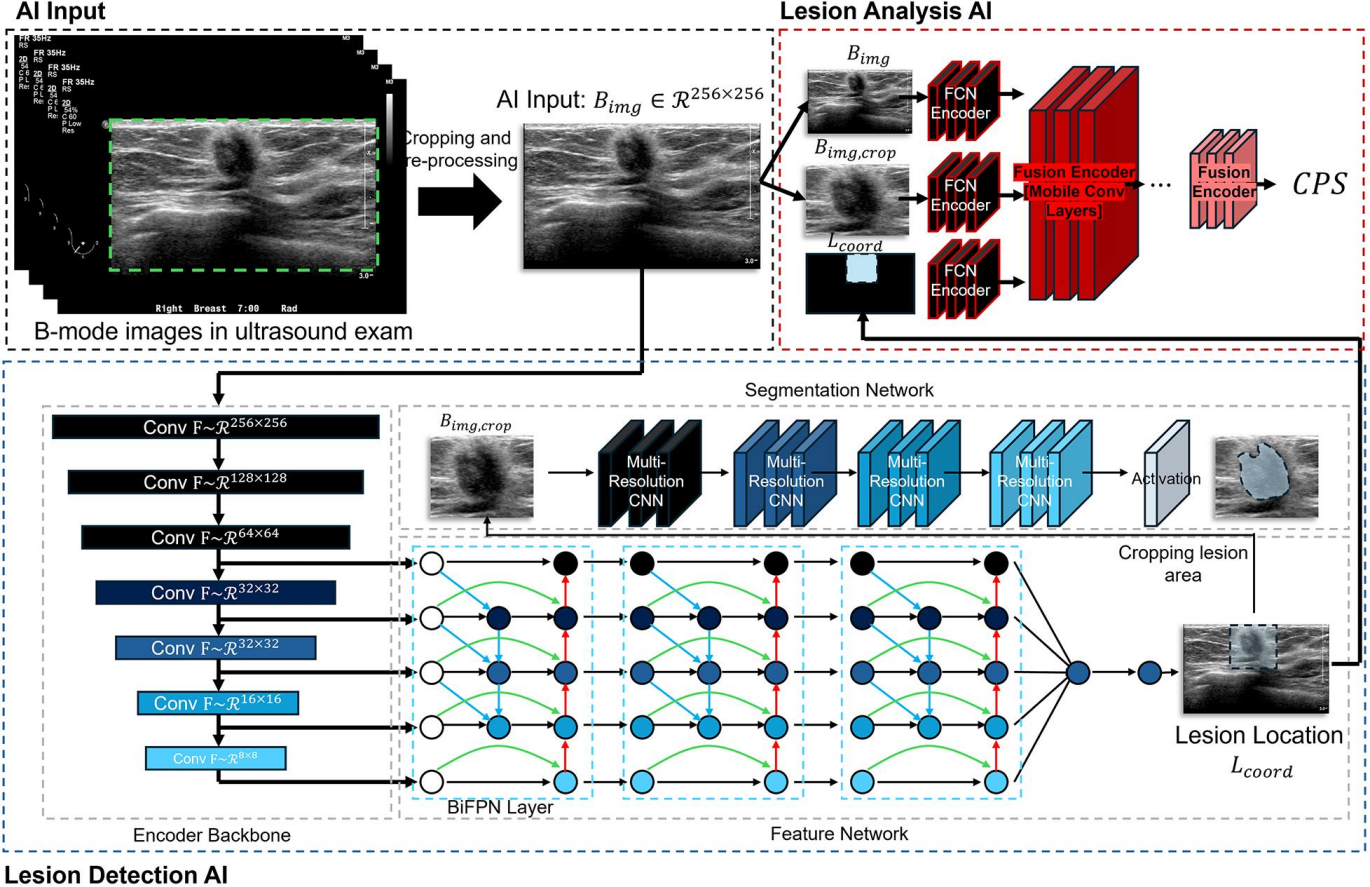
Overall configuration of the Vis-BUS neural networks. Vis-BUS integrates two primary components: Lesion Detection AI (LD-AI) and Lesion Analysis AI (LA-AI). LD-AI uses b-mode ultrasound images, $B_{\mathrm{img}}\;∼\;R^{256X256}$, to identify the location of the lesion, while LA-AI analyzes the breast b-mode image characteristics.

This output is reported as the Cancer Probability Score (CPS; −100 = definitely benign to +100 = definitely malignant), a continuous malignancy confidence score that conveys the algorithm's graded assessment. When applied clinically, a positive CPS (>0) is intended to prompt consideration of biopsy, while the full −100 to +100 range supports ROC-based discrimination analysis. A probabilistic score allows clinicians to adjust decision thresholds based on patient context and risk tolerance, facilitates nuanced discussions with patients, and supports downstream triage or follow-up strategies. The CPS is defined on a symmetric −100 to +100 scale to center the decision boundary at 0, aligning with a “no-biopsy vs. biopsy” dichotomy. This symmetric scaling improves interpretability for up- or down-titrating thresholds (e.g., screening vs. diagnostic contexts) and mirrors the sign of the model's log-odds while preserving a familiar 200-point visual range. Models were optimized using AdamW with typical learning rates on the order of 10^−4^, and standard data-augmentation and early-stopping strategies were applied to improve generalization (Figure 1). Additional details on dataset composition, exact train–validation–test splits, specific hyperparameters and training protocols are provided in the Supplementary Material.

### Assistance protocol

Each radiologist conducted two reading sessions separated by a two-week washout. In Session 1 (unaided) the reader, blinded to clinical and pathologic data, reviewed every case, decided recall vs. no-recall, drew an ROI box around the lesion, assigned a cancer-probability score (CPS; −100 = definitely benign to +100 = definitely malignant), and the workstation automatically logged interpretation time. In Session 2 (AI-assisted) the same 258 cases were re-randomized; Vis-BUS displayed its bounding box and malignancy likelihood, after which the reader could adjust the ROI and CPS following the identical workflow while interpretation time was again recorded.

Two complementary endpoints were collected per case: (1) a binary action decision (recall vs. no-recall), representing the reader's clinical recommendation for biopsy or further workup; and (2) a continuous Cancer Probability Score (CPS; −100 = definitely benign to +100 = definitely malignant), used for ROC-based discrimination analysis. Sensitivity and specificity were derived from the binary recall decision, while AUROC was derived from the continuous CPS. The ground truth was the definitive diagnosis: pathology-confirmed malignancy, biopsy-proven benign, or benign by stable imaging follow-up (≥12 months).

### Outcome measures

The primary outcomes were diagnostic discrimination metrics, including the area under the receiver operating characteristic curve (AUROC) and the area under the precision–recall curve (AUPRC), comparing unaided and AI-assisted readings.

The secondary outcomes included overall diagnostic accuracy, sensitivity, specificity, positive predictive value (PPV), negative predictive value (NPV), interpretation time per case, and inter-reader agreement. Subgroup analyses were conducted according to patient age (< 50 years vs. ≥ 50 years), breast density (dense vs. non-dense), tumor size (≤ 2 cm vs. > 2 cm), and lesion morphology. Lesion size was measured as the maximal diameter on B-mode ultrasound for all cases. Pathologic tumor size was available for surgically resected malignant cases and was used for T-stage classification.

Microcalcification-dominant lesions were defined as US-visible lesions in which echogenic foci consistent with mammographic microcalcifications constituted the predominant imaging feature, with or without an accompanying mass; classification was determined by the consensus of two radiologists based on paired mammogram-US review.

### Statistical analysis

Statistical analyses were performed using R (version 4.5). Multi-reader multi-case analysis of variance was applied using the Obuchowski–Rockette framework, treating readers and cases as random effects. Differences in AUROC and AUPRC were assessed with paired comparisons, while accuracy, sensitivity, specificity, PPV, and NPV were analyzed with generalized linear mixed models. Reading times were compared using the Wilcoxon signed-rank test.

In this reader study, sensitivity was defined as the proportion of pathology-confirmed malignancies for which the reader recommended recall, and specificity as the proportion of confirmed benign lesions for which the reader recommended no recall. These definitions reflect each reader's independent, blinded assessment of pre-selected cases and are standard in MRMC reader study methodology (16).

Both continuous CPS data (for AUROC) and binary recall decisions (for sensitivity/specificity) were collected from each reader for every case. This dual-endpoint approach is standard in MRMC reader studies and is explicitly endorsed by the FDA guidance for CAD clinical performance assessment (2022). The continuous CPS enables full ROC curve estimation across all possible thresholds, while the binary recall decision provides a clinically interpretable operating point. These complementary endpoints are analyzed with separate statistical methods and answer distinct questions about discriminative ability vs. point-estimate diagnostic accuracy (17).

Our sample size determination followed the recommended hybrid approach: all eligible consecutive malignant cases from the accrual window (2015–2020) were enumerated (*n* = 129), and benign cases were randomly sampled to achieve a 1:1 ratio (*n* = 129), yielding 258 total cases. An *a priori* power calculation using conjectured variance parameters from published MRMC studies confirmed that a minimum of 239 cases was required to detect an absolute AUROC increase of 0.03 with 85% power at a two-sided *α* of 0.05 (15). The final cohort of 258 cases exceeded this requirement, providing a buffer for potential case exclusion. Key statistical definitions and formulas are summarized in Supplementary Material S3 (Supplementary Table S2).

## Results

### Patient population and case characteristics

**Figure 2:**
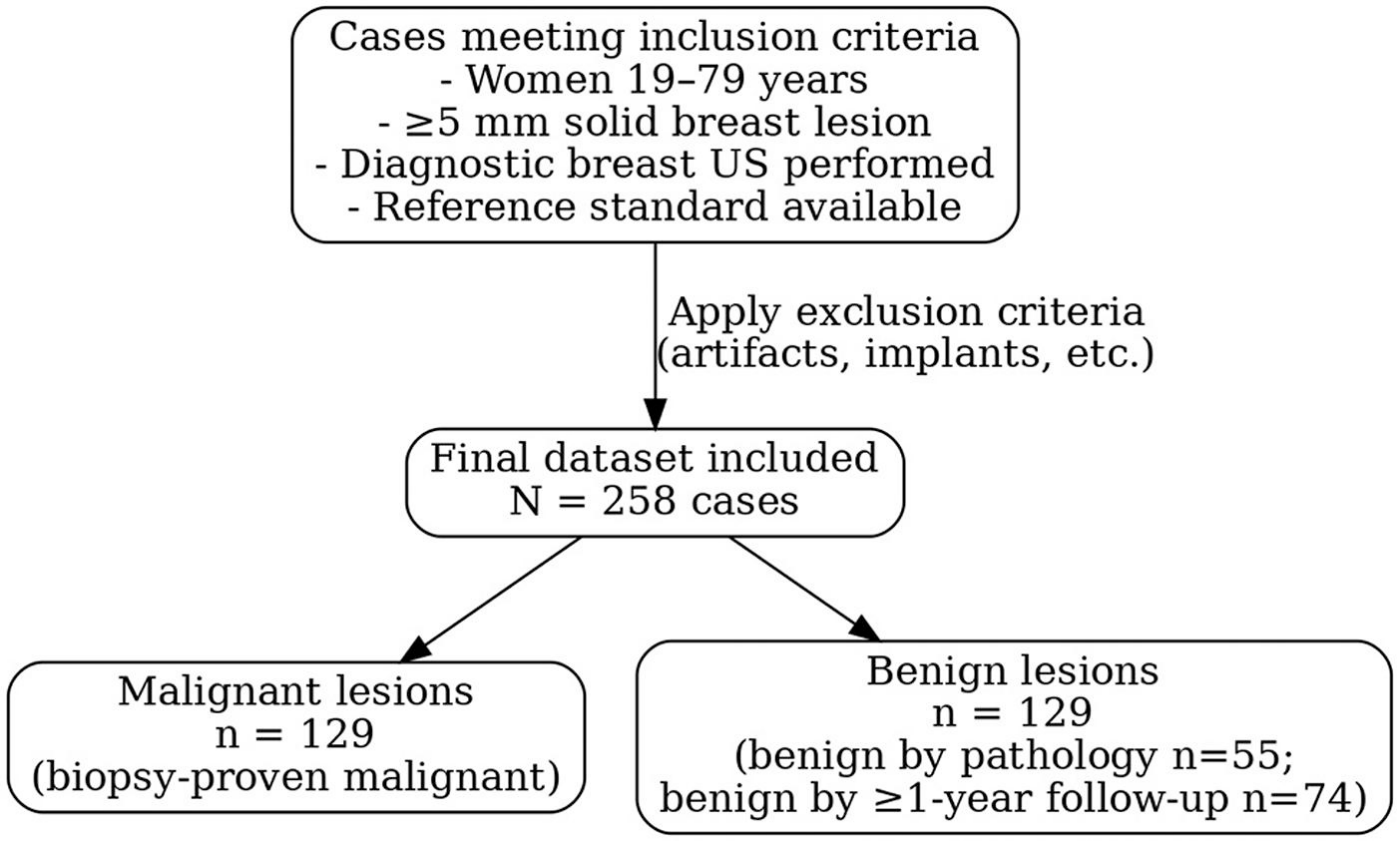
Case enrollment. Flowchart illustrating the retrospective case selection process and lesion classification pipeline. After applying the inclusion criteria and then excluding cases that met any exclusion criteria, a final cohort of 258 eligible ultrasound cases was obtained. The final dataset comprised 129 malignant cases and 129 benign cases. Malignant lesions were all confirmed as cancers by biopsy, whereas benign lesions were confirmed either by benign pathology or by stable imaging findings over ≥1 year. An enrichment sampling strategy was used to ensure equal numbers of malignant and benign cases in the study cohort.

**Figure 3:**
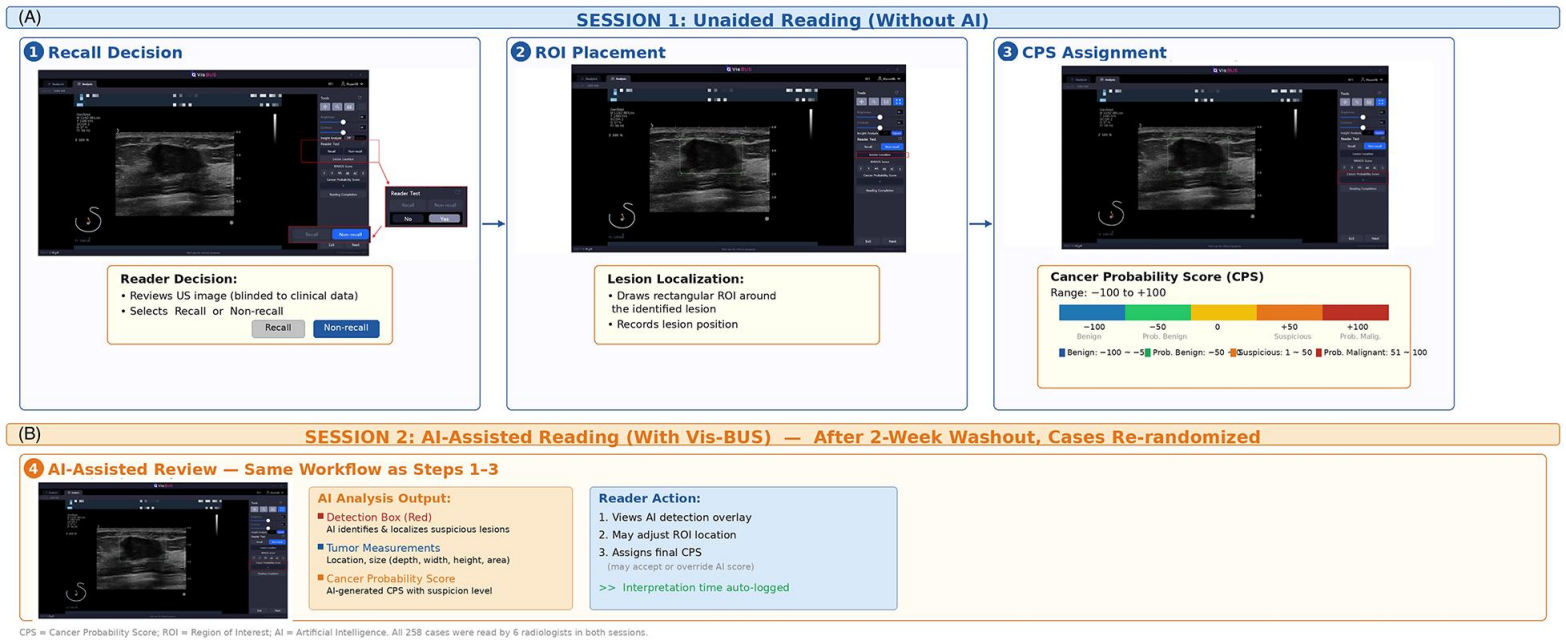
Vis-BUS–assisted versus unaided reader assessments in breast ultrasound **(A)** session 1 (unaided. 1-3): Radiologists, blinded to clinical and pathologic data, reviewed each of the 258 cases, decided “recall” versus “no recall,” drew a rectangular region of interest (ROI) around the lesion, and assigned a cancer probability score (CPS; –100 = definitely benign to +100 = definitely malignant). Interpretation time for each case was automatically recorded by the workstation. **(B)** Session 2 (AI-Assisted, 4): After a two-week washout, the same cases were re-randomized. Vis-BUS automatically overlaid its bounding box indicating lesion location and displayed a malignancy likelihood score. Readers then adjusted the ROI and CPS following the identical workflow, with interpretation time again logged.

A total of 258 breast ultrasound cases (129 malignant and 129 benign) from 258 patients were included (Figure 2) and each radiologist finished two reading sessions separated by a two-week washout (Figure 3). Patient age ranged from 20 to 78 years (mean 48.2 ± 11.0; median 47 years). Malignant cases were on average older than benign cases (median age 50 vs. 46 years, *p* < .001). Most patients (196, 76.0%) had dense breasts on mammography. Prior breast lesion or cancer history was present in 34 cases (13.2%). Patients with a remote history of breast cancer were eligible if they were not undergoing active cancer treatment at the time of imaging and if the index lesion in this study had an independent reference standard. Cases with post-procedural imaging (after vacuum-assisted biopsy or surgical excision) were excluded per protocol. This clarifies why 34 cases had a prior history while still meeting the prespecified exclusion criteria. All 129 malignancies were confirmed by pathology (112 invasive cancers and 17 ductal carcinoma *in situ*), while of the 129 benign lesions, 55 (42.6%) were biopsy-proven benign and 74 (57.4%) were confirmed stable on ≥1-year imaging follow-up (BI-RADS 2 or 3 outcome). Lesion size ranged from 0.5 to 8.5 cm (median, 1.3 cm); malignant lesions were larger on average (median 2.0 cm vs. 0.9 cm for benign). By T stage, 70/129 (54.3%) cancers were ≤2 cm, 48 (37.2%) were 2–5 cm, and 11 (8.5%) exceeded 5 cm. Sonographic morphology differed markedly between benign and malignant lesions: 96.1% of cancers appeared irregular in shape (vs. 51.2% of benign lesions), whereas most benign lesions were oval (45.0% vs. 3.1% of cancers). Margins were predominantly indistinct (68.2% overall), with spiculated margins in 25/129 cancers (19.4%) and 1/129 benign lesions (0.8%) Sonographic morphology descriptors (shape, margin, echogenicity) were abstracted from the original clinical ultrasound reports and are presented in Table 1.

**Table 1:** Patient and case characteristics.

Characteristic	All patients (*N* = 258)	Benign (*n* = 129)	Malignant (*n* = 129)	*P*-value
Age (years)	48.2 ± 11.0 (20–78)	44.9 ± 10.0 (20–78)	51.6 ± 10.9 (22–78)	<.001
Lesion size (cm)	1.7 ± 1.4, median 1.3 [0.8–2.1]	1.0 ± 0.6, median 0.9 [0.6–1.2]	2.4 ± 1.6, median 2.0 [1.4–3.0]	<.001
Breast density, *n* (%)				<.001
ACR A	2 (0.8%)	0 (0.0%)	2 (1.6%)	
ACR B	25 (9.7%)	3 (2.3%)	22 (17.1%)	
ACR C	148 (57.4%)	78 (60.5%)	70 (54.3%)	
ACR D	48 (18.6%)	26 (20.2%)	22 (17.1%)	
Unknown	35 (13.6%)	22 (17.1%)	13 (10.1%)	
Lesion shape, *n* (%)				<.001
Oval	62 (24.0%)	58 (45.0%)	4 (3.1%)	
Round	6 (2.3%)	5 (3.9%)	1 (0.8%)	
Irregular	190 (73.6%)	66 (51.2%)	124 (96.1%)	
Margin, *n* (%)				<.001
Circumscribed	43 (16.7%)	41 (31.8%)	2 (1.6%)	
Indistinct	176 (68.2%)	85 (65.9%)	91 (70.5%)	
Angular	13 (5.0%)	2 (1.6%)	11 (8.5%)	
Spiculated	26 (10.1%)	1 (0.8%)	25 (19.4%)	
Echogenicity, *n* (%)				<.001
Hypoechoic	210 (81.4%)	96 (74.4%)	114 (88.4%)	
Isoechoic	28 (10.9%)	25 (19.4%)	3 (2.3%)	
Heterogeneous	15 (5.8%)	6 (4.7%)	9 (7.0%)	
Complex/Hyperechoic	5 (1.9%)	2 (1.6%)	3 (2.3%)	
BI-RADS category, *n* (%)				<.001
C2	35 (13.6%)	35 (27.1%)	0 (0.0%)	
C3	48 (18.6%)	48 (37.2%)	0 (0.0%)	
C4	69 (26.7%)	46 (35.7%)	23 (17.8%)	
C5	106 (41.1%)	0 (0.0%)	106 (82.2%	

Age is given as mea*n* ± SD with range in parentheses; lesion size as mean ± SD with median [IQR]; categorical variables are number of patients, with percentages in parentheses.

ACR, American College of Radiology; BI-RADS, Breast Imaging Reporting and Data System; IQR, interquartile range; SD, standard deviation; cm, centimeter.

### Reader accuracy and diagnostic performance

Interpretation time was defined as the elapsed time from case display to submission of the recall decision and CPS score, automatically logged by the workstation. This interval encompasses the complete per-case reading task (image review, recall decision, ROI placement, and CPS assignment) but does not include clinical scanning, mammographic correlation, or report generation.

**Figure 4:**
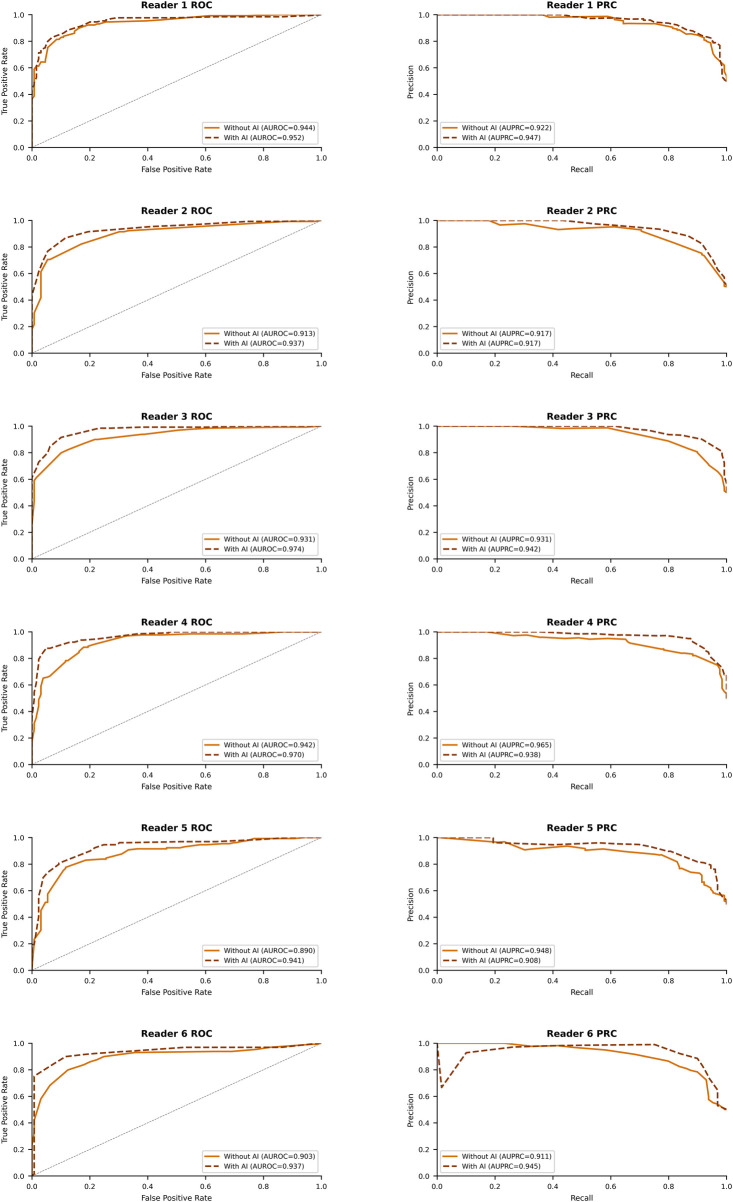
Receiver operating characteristic (ROC) and precision–recall (PR) curves for each reader and the pooled analysis, comparing unaided readings (solid lines) with Vis-BUS AI–assisted readings (dashed lines). In the pooled analysis, the AUROC rose from 0.921 (95% CI, 0.889–0.954) without AI to 0.953 (95% CI, 0.930–0.975; *Δ* = 0.031; *p* = 0.002), while the AUPRC increased from 0.932 (95% CI, 0.921–0.943) to 0.933 (95% CI, 0.923–0.942; *Δ* = 0.001; *p* = 0.944). Reader 1's AUROC/AUPRC changed from 0.944/0.922 to 0.952/0.947 (*p* = 0.339/0.495); Reader 2's from 0.913/0.917 to 0.937/0.917 (*p* = 0.078/0.965); Reader 3's from 0.931/0.931 to 0.974/0.942 (*p* < .001/0.546); Reader 4's from 0.942/0.965 to 0.970/0.938 (*p* = 0.008/0.478); Reader 5's from 0.890/0.948 to 0.941/0.908 (*p* = 0.003/0.531); and Reader 6's from 0.903/0.911 to 0.937/0.945 (*p* = 0.081/0.493).

All six radiologists demonstrated improved diagnostic discrimination with Vis-BUS AI assistance (Table 2). At the pooled level, the area under the ROC curve (AUROC) increased from 0.921 (95% CI 0.889–0.954) without AI to 0.953 (95% CI 0.930–0.975)—a gain of 0.032 (95% CI 0.013–0.050; *p* = 0.002). In the pooled analysis, AUPRC was 0.932 without AI vs. 0.933 with AI; this difference was not statistically significant (*Δ* = 0.001; 95% CI −0.013 to 0.015; *p* = 0.944) (Figure 4).

**Table 2:** Per-Reader diagnostic performance without vs With AI assistance.

Reader (year of experience)	Metric	Without AI (95% CI)	With AI (95% CI)	Δ (95% CI)	*p*-value
Pooled	AUROC	0.921 (0.889–0.954)	0.953 (0.930–0.975)	+0.032 (0.013–0.050)	0.002
	AUPRC	0.932 (0.921- 0.943)	0.933 (0.923- 0.942)	+0.001 (−0.013- 0.015)	0.944
	Sensitivity	94.2% (90.6–96.5)	96.3% (93.5–97.9)	+2.1% (–1.6–5.6)	0.243
	Specificity	64.0% (57.9–69.6)	71.6% (65.5–76.9)	+7.6% (–0.5–15.8)	0.069
	PPV	58.4% (51.8–64.7)	63.9% (57.1–70.2)	+5.5% (–3.7–14.8)	0.243
	NPV	86.3% (80.1–90.9)	90.8% (85.1–94.4)	+4.5% (–2.6–11.5)	0.219
	Accuracy	79.1% (75.1–82.6)	83.9% (80.2–87.0)	+4.8% (–2.4–11.9)	0.061
Reader 1 (17)	AUROC	0.944 (0.918–0.970)	0.952 (0.926–0.978)	+0.008 (–0.044–0.060)	0.339
	AUPRC	0.922 (0.895-0.945)	0.947 (0.927-0.965)	+0.025 (−0.003-0.057)	0.495
	Sensitivity	90.7% (84.3–95.1)	96.9% (92.3–99.1)	+6.2% (–2.8–14.8)	0.013
	Specificity	75.2% (66.8–82.4)	63.6% (54.6–71.9)	–11.6% (–27.8–5.1)	0.005
	PPV	78.5% (71.1–84.8)	72.7% (65.4–79.2)	–5.8% (–19.4–8.1)	0.015
	NPV	89.0% (81.6–94.2)	95.3% (88.5–98.7)	+6.3% (–5.7–17.1)	0.019
	Accuracy	82.9% (77.8–87.3)	80.2% (74.8–84.9)	–2.7% (–12.5–7.1)	0.296
Reader 2 (1)	AUROC	0.913 (0.878–0.948)	0.937 (0.908–0.966)	+0.024 (–0.040–0.088)	0.078
	AUPRC	0.917 (0.890-0.942)	0.917 (0.888-0.942)	+0.001 (−0.039-0.038)	0.965
	Sensitivity	96.9% (92.3–99.1)	94.6% (89.1–97.8)	–2.3% (–10.0–5.5)	0.45
	Specificity	51.9% (43.0–60.8)	72.9% (64.3–80.3)	+21.0% (3.5–37.3)	<.001
	PPV	66.8% (59.6–73.5)	77.7% (70.4–84.0)	+10.9% (–3.1–24.4)	<.001
	NPV	94.4% (86.2–98.4)	93.1% (86.2–97.2)	–1.3% (–12.2–11.0)	0.66
	Accuracy	74.4% (68.6–79.6)	83.7% (78.6–88.0)	+9.3% (–1.0–19.4)	<.001
Reader 3 (14)	AUC	0.931 (0.902–0.960)	0.974 (0.960–0.989)	+0.043 (0.000–0.087)	<.001
	AUPRC	0.931 (0.903-0.953)	0.942 (0.920-0.960)	+0.011 (−0.018-0.043)	0.546
	Sensitivity	91.5% (85.3–95.7)	96.1% (91.2–98.7)	+4.6% (–4.5–13.4)	0.114
	Specificity	73.6% (65.2–81.0)	81.4% (73.6–87.7)	+7.8% (–7.4–22.5)	0.034
	PPV	77.6% (70.2–84.0)	83.8% (76.8–89.3)	+6.2% (–7.2–19.1)	0.008
	NPV	85.4% (80.3–89.5)	86.6% (81.8–90.5)	+1.2% (–7.7–10.2)	0.604
	Accuracy	82.6% (77.4–87.0)	88.8% (84.3–92.3)	+6.2% (–2.7–14.9)	0.005
Reader 4 (38)	AUC	0.942 (0.916–0.969)	0.970 (0.953–0.987)	+0.028 (–0.016–0.071)	0.008
	AUPRC	0.965 (0.949-0.977)	0.938 (0.915-0.959)	-0.027 (−0.052-0.002)	0.478
	Sensitivity	95.3% (90.2–98.3)	97.7% (93.4–99.5)	+2.4% (–4.9–9.3)	0.450
	Specificity	76.0% (67.7–83.1)	72.9% (64.3–80.3)	–3.1% (–18.8–12.6)	0.556
	PPV	79.9% (72.7–85.9)	78.3% (71.1–84.4)	–1.6% (–14.8–11.7)	0.533
	NPV	88.2% (83.5–92.0)	86.6% (81.8–90.5)	–1.6% (–10.2–7.0)	0.462
	Accuracy	85.7% (80.8–89.7)	85.3% (80.3–89.4)	–0.4% (–9.4–8.6)	1
Reader 5 (1)	AUROC	0.890 (0.851–0.930)	0.941 (0.912–0.970)	+0.051 (–0.018–0.119)	0.003
	AUPRC	0.948 (0.913-0.974)	0.908 (0.871-0.9380	-0.04 (−0.086-0.004)	0.531
	Sensitivity	96.9% (92.3–99.1)	97.7% (93.4–99.5)	+0.8% (–5.7–7.2)	1.000
	Specificity	41.1% (32.5–50.1)	61.2% (52.3–69.7)	+20.1% (2.2–37.2)	<.001
	PPV	62.2% (55.1–68.9)	71.6% (64.3–78.1)	+9.4% (–4.6–23.0)	<.001
	NPV	88.6% (82.7–93.0)	86.6% (81.8–90.5)	–2.0% (–11.2–7.8)	0.436
	Accuracy	69.0% (63.0–74.6)	79.5% (74.0–84.2)	+10.5% (–0.6–21.2)	<.001
Reader 6 (1)	AUROC	0.903 (0.865–0.941)	0.937 (0.904–0.970)	+0.034 (–0.037–0.105)	0.081
	AUPRC	0.911 (0.880-0.937)	0.945 (0.922-0.965)	+0.035 (−0.003-0.072)	0.493
	Sensitivity	93.8% (88.1–97.3)	94.6% (89.1–97.8)	+0.8% (–8.2–9.7)	1.000
	Specificity	65.9% (57.0–74.0)	77.5% (69.3–84.4)	+11.6% (–4.7–27.4)	0.012
	PPV	73.3% (65.9–79.9)	80.8% (73.6–86.7)	+7.5% (–6.3–20.8)	0.006
	NPV	86.5% (81.5–90.6)	86.6% (81.8–90.5)	+0.1% (–8.8–9.0)	0.967
	Accuracy	79.8% (74.4–84.6)	86.0% (81.2–90.0)	+6.2% (–3.4–15.6)	0.015

AUROC, area under the receiver operating characteristic curve; AUPRC, area under the precision–recall curve; CI, confidence interval; PPV, positive predictive value; NPV, negative predictive value; Δ = absolute change (With AI minus Without AI).

Pooled sensitivity increased from 94.2% (90.6–96.5) to 96.3% (93.5–97.9), an improvement of 2.1 points (95% CI –1.6–5.6; *p* = 0.243), while pooled specificity improved from 64.0% (57.9–69.6) to 71.6% (65.5–76.9), a gain of 7.6 points (95% CI –0.5–15.8; *p* = 0.069). Positive predictive value (PPV) rose from 58.4% to 63.9% (+5.5 points; 95% CI –3.7–14.8; *p* = 0.243), negative predictive value (NPV) from 86.3% to 90.8% (+4.5 points; 95% CI –2.6–11.5; *p* = 0.219), and overall accuracy from 79.1% to 83.9% (+4.8 points; 95% CI –2.4–11.9; *p* = 0.061).

Individually, all six readers experienced AUROC gains with AI, with significant improvements in three readers (R3: 0.931→0.974, *p* < .001; R4: 0.942→0.970, *p* = 0.008; R5: 0.890→0.941, *p* = 0.003). Changes in AUPRC varied by reader (*Δ* –0.040 to +0.035) and did not reach statistical significance for any individual. Sensitivity increased in five of six readers, achieving significance only for Reader 1 (90.7%→96.9%, *p* = 0.013), while specificity improvements were significant for Readers 2 (+21.0 points, *p* < .001), 3 (+7.8 points, *p* = 0.034), 5 (+20.1 points, *p* < .001), and 6 (+11.6 points, *p* = 0.012). PPV and NPV trends mirrored these patterns, and accuracy improved significantly in four readers.

### Reader efficiency and interpretation time

**Figure 5:**
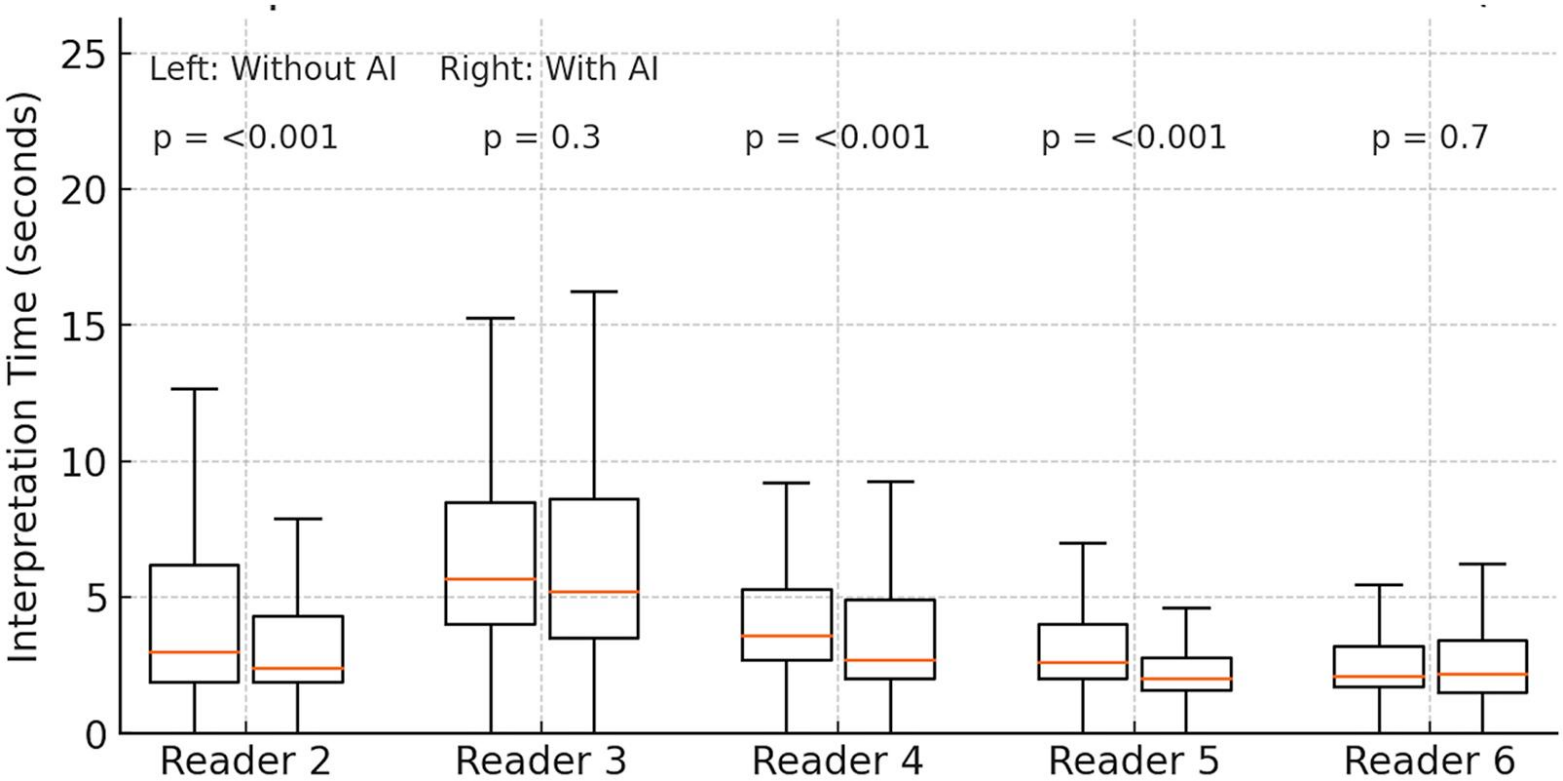
Per-Reader interpretation times without vs With AI assistance (readers 2–6). Box-and-whisker plots showing the distribution of per-case ultrasound interpretation times for five radiologists (Readers 2–6), comparing unaided readings (“Without AI,” white boxes) to AI-assisted readings (“With AI,” orange boxes). Boxes span the interquartile range (25th–75th percentiles), whiskers extend to the most extreme values within 1.5×IQR, and horizontal lines denote medians. Above each reader's pair, the *p*-value from a two-sided paired Wilcoxon signed-rank test is annotated, indicating the statistical significance of time reductions with Vis-BUS assistance. Reader 1 is omitted from this panel because its extreme range (median 9 s, IQR 4–40 s) would compress the *y*-axis and obscure detail.

AI assistance significantly improved reading efficiency for most readers. With Vis-BUS, the median interpretation time per case decreased by 1–2 s for most readers (Table 3). For example, Reader 1's median read time dropped from 9 s (IQR 4–40) without AI to 3 s (IQR 2–4) with AI (*p* < .001). Reader 4's median time went from 3.6 (2.7–5.3) to 2.7 (2.0–4.9) seconds (*p* < .001), and Reader 5 from 2.6 to 2.0 s (*p* < .001). In contrast, two readers showed no meaningful change in speed: Reader 3's median was 5.7 vs. 5.2 s with AI (*p* = 0.3), and Reader 6's times were 2.1 vs. 2.2 s (*p* = 0.7). Pooled across all readings, the median interpretation time fell from 6.0 s without AI to 3.0 s with AI (Wilcoxon *p* < .001). Mean reading time per case also declined from 28.0 ± 5.6 s to 9.5 ± 1.9 s with AI. Figure 5 illustrates the distribution of case reading times by condition, showing a general left-shift (faster reads) with AI assistance.

**Table 3:** Per-reader interpretation time With and without AI assistance.

Reader (year of experience)	Without AI, sec (IQR)	With AI (IQR)	Δ (95% CI)	*P*-value^†^
Reader 1 (17)	9 (4, 40)	3 (2, 4)	5.7 (3.6-8.6)	<.001
Reader 2 (1)	3.0 (1.9, 6.2)	2.4 (1.9, 4.3)	0.2 (0-0.4)	<.001
Reader 3 (14)	5.7 (4.0, 8.5)	5.2 (3.5, 8.6)	0.4 (−0.3-0.8)	0.3
Reader 4 (36)	3.6 (2.7, 5.3)	2.7 (2.0, 4.9)	0.8 (0.5–1.1)	<.001
Reader 5 (1)	2.60 (2.00, 4.00)	2.00 (1.60, 2.80)	0.5 (0.4–0.7)	<.001
Reader 6 (1)	2.10 (1.70, 3.20)	2.20 (1.50, 3.40)	0.2 (0–0.4)	0.7

CI = confidence interval, IQR=interquartile range

† *P*-values from paired two-sided Wilcoxon signed-rank tests (comparing unaided vs AI-assisted conditions).

### Subgroup performance and error patterns

Radiologist performance improved markedly with AI support in specific challenging subgroups (Table 4). For small tumors (T1 ≤ 2 cm), AI assistance increased the average reader AUROC from 0.889 without AI to 0.933 with AI (*Δ* = 0.044; *p* < 0.001).

**Table 4:** Diagnostic performance across subgroups With and without AI.

Sub-group	Without AI	With AI	Δ95 % CI	*P*-value
AUROC				
Age < 50 y	0.906	0.946	+0.041 (0.017–0.065)	0.002
Age ≥ 50 y	0.911	0.953	+0.043 (0.014–0.073)	0.004
Dense breasts	0.902	0.944	+0.042 (0.021–0.064)	<.001
Calcifications present	0.911	0.956	+0.045 (0.008–0.085)	0.012
Calcifications absent	0.912	0.947	+0.035 (0.012–0.059)	0.002
Tumor size ≤ 2 cm	0.889	0.933	+0.044 (0.019–0.070)	<.001
Tumor size 2–5 cm	0.891	0.952	+0.060 (–0.002–0.129)	0.062
Irregular shape	0.891	0.942	+0.050 (0.028–0.073)	<.001
General radiologists¹	0.922	0.955	+0.034 (0.010–0.057)	0.003
Breast-imaging specialists¹	0.904	0.946	+0.042 (0.015–0.070)	0.002
Sensitivity				
Age < 50 y	93.1	96	+2.9 (–2.3 to 8.1)	0.263
Age ≥ 50 y	95.2	96.5	+1.3 (–3.5 to 6.0)	0.599
Dense breasts	93.5	95.5	+2.0 (–2.2 to 6.2)	0.346
Calcifications present	94.4	96.7	+2.3 (–2.3 to 6.8)	0.311
Calcifications absent	93.9	95.8	+1.9 (–3.6 to 7.3)	0.500
Tumor size ≤ 2 cm	92.1	94	+1.9 (–3.7 to 7.5)	0.502
Tumor size 2–5 cm	96.2	99	+2.8 (–1.7 to 7.3)	0.159
Irregular shape	94.3	96.1	+1.9 (–1.8 to 5.5)	0.304
General radiologists¹	94.8	95.7	+1.0 (–2.8 to 4.7)	0.611
Breast-imaging specialists¹	93	97.3	+4.3 (–0.2 to 8.7)	0.060
Specificity				
Age < 50 y	64.8	72	+7.3 (–2.3 to 16.9)	0.140
Age ≥ 50 y	61.6	70.2	+8.6 (–6.8 to 24.0)	0.277
Dense breasts	65.7	73.1	+7.4 (–1.8 to 16.5)	0.117
Calcifications present	66.7	70.5	+3.8 (–23.7 to 31.4)	0.784
Calcifications absent	63.6	71.7	+8.0 (–0.5 to 16.6)	0.067
Tumor size ≤ 2 cm	64.9	72.5	+7.6 (–0.7 to 15.9)	0.074
Tumor size 2–5 cm	44.4	52.8	+8.3 (–31.0 to 47.7)	0.679
Irregular shape	38.9	52	+13.1 (–3.9 to 30.1)	0.134
General radiologists¹	58.1	73.3	+15.1 (6.6 to 23.6)	0.001
Breast-imaging specialists¹	75.6	68.2	–7.4 (–16.5 to 1.8)	0.118

AUROC, area under the receiver operating characteristic curve, CI = confidence interval.

AI assistance significantly enhanced diagnostic performance across challenging subgroups and improved both sensitivity and specificity. In dense breasts (BI-RADS C/D), the AUROC increased from 0.902 to 0.944 (*Δ* = 0.042; *p* < .001), sensitivity rose from 93.5% to 95.5% (*Δ* = 2.0%; *p* = 0.346), and specificity improved from 65.7% to 73.1% (*Δ* = 7.4%; *p* = 0.117). For microcalcification-dominant lesions, the AUROC increased from 0.911 to 0.956 (*Δ* = 0.045; *p* = 0.012), sensitivity from 94.4% to 96.7% (*Δ* = 2.3%; *p* = 0.311), and specificity from 66.7% to 70.5% (*Δ* = 3.8%; *p* = 0.784). Gains in easier subgroups (e.g., larger tumors, non-dense tissue) were minimal and not statistically significant (*p* > 0.05). AI also narrowed the gap between reader types: general radiologists' AUROC rose from 0.922 to 0.955 (*Δ* = 0.034; *p* = 0.003) and breast imaging specialists from 0.904 to 0.946 (*Δ* = 0.042; *p* = 0.002), reducing the inter-group difference to just 0.009 (*p* = NS). Sensitivity for general radiologists improved from 94.8% to 95.7% (*Δ* = 1.0%; *p* = 0.611), and specificity increased markedly from 58.1% to 73.3% (*Δ* = 15.1%; *p* = 0.001).

Error pattern analysis compared the standalone AI binary classification (CPS > 0 = positive) against unaided reader performance. Of the 33 cases misclassified by the standalone AI, 12 were cancers that the AI failed to detect (AI false-negatives) but that were correctly recalled by at least one of the six unaided readers. Conversely, 5 cancers were correctly flagged by the standalone AI (CPS > 0) but missed by all six unaided readers (i.e., all six decided “no recall”). This complementary error pattern—in which AI and human readers tended to miss different subsets of cancers—underscores the synergistic benefit of combining AI assistance with radiologist interpretation to maximize overall detection (Table 5).

**Table 5:** Radiologist behavior in cases misclassified by AI alone (matching result=N): reader accuracy distribution and Wilcoxon signed-rank analysis.

No. of Readers Correct per Case	Unaided Reading (no AI) No. of cases (%)	AI-assisted Reading No. of cases (%)
0	7 (21%)	8 (24%)
1	5 (15%)	7 (21%)
2	4 (12%)	5 (15%)
3	4 (12%)	4 (12%)
4	4 (12%)	5 (15%)
5	5 (15%)	2 (6%)
6	4 (12%)	2 (6%)
Median [IQR] correct readers per case	3 [1–5]	2 [1–4]
Wilcoxon signed-rank test†	**z** **=** **–2.99**	***p*** **=** **0.003**

IQR, interquartile range.

Data are based on 33 cases in which the AI system (Vis-BUS) misclassified the lesion (matching_result=N). Values in parentheses are percentages of these 33 cases. This is a per-case paired comparison of reader performance without vs with AI assistance (Vis-BUS). The Wilcoxon signed-rank test compares the number of correct readers (out of six) per case between conditions.

The AI assistance reduced variability among radiologists – the intraclass correlation coefficient for inter-reader agreement increased from moderate reliability (ICC = 0.70; 95% CI 0.65–0.75) to good reliability (ICC = 0.78; 95% CI 0.74–0.82), reflecting more consistent interpretations.

**Figure 6:**
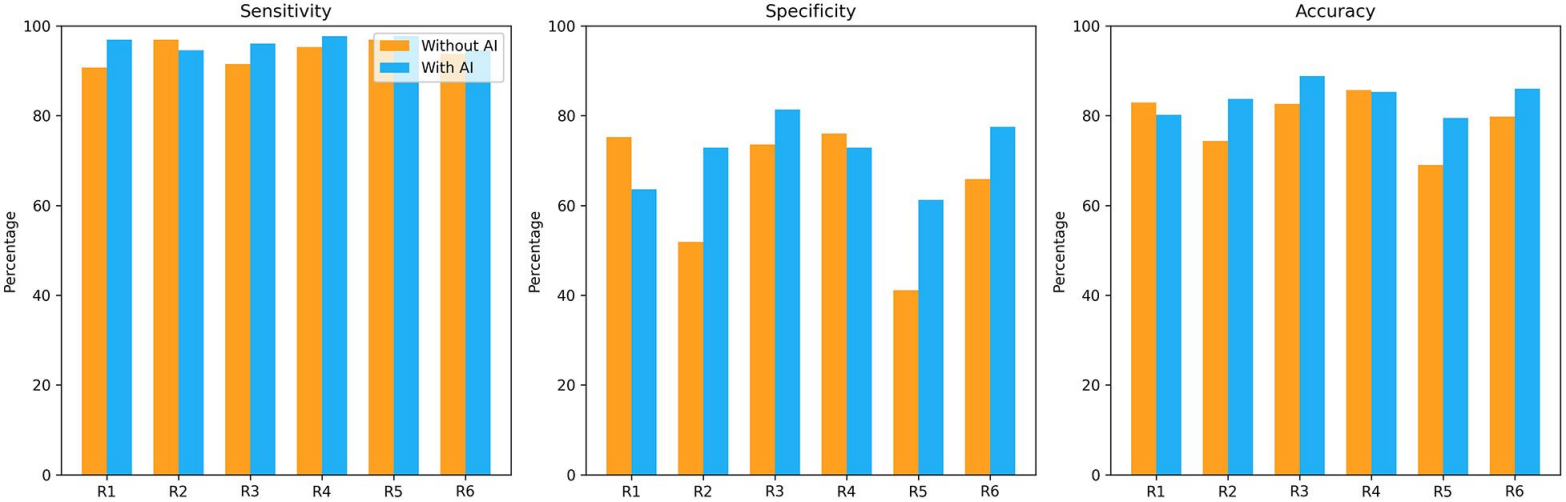
Effect of AI on diagnostic performance and efficiency. Bar charts summarize sensitivity, specificity and overall accuracy for each reader in unaided (yellow) and AI-assisted (orange) conditions. Across all metrics, Vis-BUS either improves or maintains performance relative to unaided readings.

Figure 6 illustrates how Vis-BUS AI affects diagnostic performance and reading efficiency for each reader. For both pooled and individual data, bar charts display sensitivity, specificity and accuracy with and without AI. Calibration analysis showed that the AI tended to overestimate malignancy probabilities at higher CPS values (Supplementary Figure S2). Detailed calibration analysis is provided in Supplementary Material S4. When using a CPS threshold of +20 to recommend biopsy, sensitivity and specificity were 91.5% and 68.2%, respectively, which approximates BI-RADS 4A recommendations.

By expertise, AUROC increased from 0.904 to 0.946 in specialists (*Δ* 0.042; *p* = 0.002) and from 0.922 to 0.955 in general radiologists (*Δ* 0.034; *p* = 0.003), narrowing the gap between groups. R3 (specialist, 14 y), R4 (general, 38 y), and R5 (general, 1 y) showed significant individual AUROC gains.

## Discussion

In this retrospective multi-reader study, use of Vis-BUS, a commercial AI breast ultrasound detection and analysis software, was associated with a statistically significant improvement in AUROC but no significant differences in AUPRC, accuracy, sensitivity, or specificity between unaided and AI-assisted interpretations. These findings suggest that while AI may aid in lesion-level discrimination, its overall diagnostic impact in terms of clinical accuracy remains limited. This interpretation is consistent with previous work showing that supplemental US enhances cancer detection in dense breasts but with increased false positives and reduced specificity (2, 3).

The reduction in median interpretation time from 6.0 to 3.0 s was statistically significant. However, the clinical impact of this finding requires caution. Time savings in image reading alone may not substantially influence the overall workflow, as the major time components in breast ultrasound include scanning the patient and drafting the report. Still, in high-volume practice settings, cumulative reductions across thousands of cases could produce meaningful efficiency gains, particularly in screening or outpatient environments (7, 18).

Subgroup analysis highlighted improved diagnostic discrimination for dense breasts and small tumors (≤2 cm). These are clinically important contexts, since dense tissue can obscure lesions on mammography and small cancers are often challenging to detect. Similar benefits of AI support in difficult diagnostic scenarios have been reported, showing increased radiologist performance when interpreting subtle lesions (12, 19).

Another notable finding was improved inter-reader consistency with AI assistance. This aligns with prior reports that AI support can reduce variability in BI-RADS classification and improve agreement among radiologists with varying levels of experience (9, 20). Such effects may be especially beneficial for less-experienced readers, narrowing the performance gap with experts and supporting training and education (9). Nevertheless, given that accuracy, sensitivity, and specificity did not differ significantly, the equalizing role of AI should be considered preliminary.

From a broader perspective, AI tools for breast imaging have demonstrated variable impact across modalities. In mammography, large-scale studies such as MASAI have confirmed that AI-supported reading can achieve non-inferior performance compared to double reading (10). Real-world nationwide implementations also support feasibility and safety of AI deployment (9). For ultrasound, feasibility studies have shown AI can function in real time to support detection and diagnosis (10), yet consistent evidence of substantial diagnostic benefit remains limited. Furthermore, questions regarding interpretability, integration with PACS, and clinician trust remain critical to adoption (8, 21). External validations and prospective trials are required to demonstrate robustness across institutions and populations (22).

The observed AUROC improvement of 0.031 was statistically significant, consistent with our *a priori* power target of detecting an absolute increase ≥0.03. However, the clinical significance of such a small absolute improvement warrants careful consideration. Prior methodological work suggests that AUROC improvements of this magnitude, while statistically detectable with adequate sample sizes, may not reliably translate to meaningful changes in patient outcomes such as recall rates, biopsy yields, or cancer detection rates (15, 23). Prospective studies with patient-level outcome endpoints are needed to determine whether the observed discrimination improvement translates to clinical benefit.

This study has several limitations. First, it was retrospective and conducted at a single institution, which limits generalizability. Second, the dataset was enriched with equal numbers of malignant and benign lesions, differing from real-world prevalence and potentially inflating diagnostic metrics. Third, only static B-mode images were analyzed; as breast ultrasound is dynamic, some clinically relevant findings may not have been captured. Fourth, in cases with multiple lesions, only an index lesion was selected, which does not reflect the complexity of multifocal disease. Fifth, the fixed reading order (unaided first, then AI-assisted) may introduce order effects, although this sequential design is supported by MRMC methodology literature (24, 25) and FDA guidance (2022) as providing greater statistical power while minimizing intra-reader variability. The unaided-first order prevents contamination of baseline performance by prior AI exposure. Although the two-week washout period and case re-randomization were intended to minimize recall bias, we cannot exclude the possibility that radiologists subconsciously remembered certain lesions. The counterbalanced crossover design suggested by the Reviewer would provide additional protection against memory effects and should be considered in future studies. Sixth, our sample size was calculated to detect an AUROC increase of 0.03, which, while statistically valid, may have limited clinical meaning. Seventh, this study was performed in a controlled laboratory setting using static B-mode images archived in PACS, rather than during real-time clinical scanning. This is a fundamental limitation inherent to all retrospective breast ultrasound reader studies (18). In clinical practice, radiologists perform dynamic scanning with real-time transducer manipulation, assess lesion compressibility and mobility, and integrate palpation findings and mammographic correlation—none of which are captured in static images. The measured interpretation times (median 3–6 s per image) reflect the image review component only and should not be extrapolated to total clinical examination time, which typically requires 10–15 min including scanning, correlation, and reporting. The clinical significance of per-image time savings can only be assessed in prospective real-time studies, where AI may provide its greatest value through concurrent decision support during scanning. Prospective, multi-center studies with real-time AI integration are needed to validate the clinical applicability of these findings. Finally, an alternative study design in which readers are randomly assigned to two independent groups—one interpreting all cases without AI and the other interpreting all cases with AI—would eliminate memory bias entirely by removing repeated exposure to the same cases. Such a parallel-group (between-reader) design avoids the carryover and recall confounders inherent in within-reader crossover studies, although it requires a larger reader panel to achieve equivalent statistical power and introduces between-group variability as a potential confounder (26, 27). Future investigations should consider this approach to provide more robust evidence of AI-assisted diagnostic benefit.

In conclusion, Vis-BUS AI assistance improved diagnostic discrimination and reduced interpretation time, while accuracy, sensitivity, and specificity did not differ significantly. These findings support AI as a potential adjunct to radiologist interpretation in breast ultrasound, but further validation in prospective, multi-center trials is required before widespread adoption.

## Data Availability

The raw data supporting the conclusions of this article will be made available by the authors, without undue reservation.
