# Supplementary material for: Artificial intelligence–assisted breast ultrasound: modest AUROC improvement and shorter interpretation time without significant change in diagnostic accuracy
Source: Front Radiol. 2026 Mar 18;6:1747783. doi: 10.3389/fradi.2026.1747783 (PMC13039021; doi:10.3389/fradi.2026.1747783)
Supplement: Supplementary file 1 [file Table1.docx]

**Supplementary Materials**

The Supplementary Materials for *Artificial Intelligence Assistance in Breast Ultrasound: Improved AUROC and Reduced Reading Time without Significant Changes in Accuracy, Sensitivity, or Specificity* comprise three appendices:

1. **Supplementary Material S1: Patient Eligibility and Dataset Construction**
   - Outlines retrospective case selection of 258 breast ultrasound exams (129 benign, 129 malignant) in women aged 19–79 with solid lesions ≥5 mm.
   - Defines detailed inclusion and exclusion criteria in narrative form and in Table S1.
   - Presents Figure S1 flowchart of case filtering and lesion classification.
2. **Supplementary Material S2: AI System Development**
   - Describes the architecture of Vis-BUS, comprising a Lesion Detection AI (LD-AI) and a Lesion Analysis AI (LA-AI).
   - Explains training data sources, neural network backbones (EfficientNet), loss functions, and the Cancer Probability Score (CPS) generation process.
3. **Supplementary Material S3: Statistical Analysis**
   - Details MRMC ANOVA (Obuchowski–Rockette) for primary AUROC comparisons.
   - Specifies subgroup analyses by lesion and patient characteristics using OR MRMC and GEE models for sensitivity/specificity/PPV/NPV.
   - Describes ICC calculation for inter-reader agreement, Wilcoxon paired tests with bootstrapped CIs for reading time, and IoU metrics for lesion localization.
   - Lists all software tools and R packages (MRMCaov, iMRMC, geepack, irr, boot) and key formulas in Table S2, plus Figure 1 schematic of the analysis workflow.
4. **Supplementary Material S4: Calibration analysis**
   - Reporting AUROC and AUPRC alone does not convey how the AI performs at clinically relevant operating points; calibration curves and threshold‑specific sensitivities/specificities are absent.

**Supplementary Material S1. Patient Eligibility and Dataset Construction**

We retrospectively identified breast ultrasound cases from the hospital PACS archives using an enrichment strategy to include equal numbers of malignant and benign lesions . Eligible patients were women aged 19–79 years with at least one solid breast lesion ≥5 mm who underwent diagnostic breast ultrasound, and each case had a definitive reference standard diagnosis available . A total of 258 cases met these inclusion criteria, comprising 129 malignant lesions and 129 benign lesions . Malignant lesions were confirmed by biopsy pathology, whereas benign lesions were confirmed either by benign pathology results (n = 55) or by demonstrating stable benign imaging findings over ≥1 year of follow-up (n = 74) . Detailed inclusion and exclusion criteria for case selection are summarized in Table S1 below.

| **Inclusion Criteria (all must apply)** | **Exclusion Criteria (any will exclude)** |
| --- | --- |
| **General:**  • Female patient, age 19–79 years .  • ≥5 mm solid breast lesion on ultrasound .  • Underwent diagnostic breast ultrasound (standard technique with two orthogonal imaging planes per lesion) .  • Definitive reference standard diagnosis available for the lesion (pathology or long-term imaging follow-up) .  **Benign lesions:**  • Lesion confirmed benign by pathology (benign biopsy result) , **or**  • Lesion not biopsied but stable on imaging follow-up for ≥1 year (at least one follow-up ≥12 months showing BI-RADS 2 or 3 assessment) .  **Malignant lesions:**  • Lesion confirmed malignant by pathology (malignant biopsy result) . | • Ultrasound images with severe artifacts that limit lesion interpretation .  • Presence of a breast implant on the ultrasound image .  • Patient was undergoing active cancer treatment at the time of imaging .  • Prominent post-biopsy changes (e.g., hematoma or scar) from a tissue sampling procedure performed ≤1 month prior to imaging .  • Ultrasound obtained after a vacuum-assisted biopsy or surgical excision of the lesion (post-procedural imaging) .  • Lesion too large to be fully captured in a single ultrasound image, or lesion with extensive calcifications precluding accurate evaluation .  • Any other condition that, in the investigators’ judgment, made the case unsuitable for the study . |

**1. Inclusion and Exclusion Criteria**

**Table S1: Detailed Inclusion and Exclusion Criteria for Case Selection.**

The inclusion criteria consisted of general requirements (patient age, lesion characteristics, imaging standards) and specific criteria for benign and malignant lesion classification. Exclusion criteria encompassed factors related to image quality and clinical context that could confound interpretation. Any case meeting an exclusion criterion was omitted from the study.

**Supplementary Material S2.. AI System Development**

1. Vis-BUS, Artificial Intelligence in Medical Imaging

Artificial Intelligence (AI), particularly through deep learning techniques, has revolutionized the field of medical imaging by enhancing the accuracy and efficiency of image analysis. In breast cancer detection, AI-driven tools analyze large datasets of imaging data to identify patterns and features that may indicate the presence of malignancy. These tools assist radiologists by providing quantitative assessments, reducing variability in interpretation, and potentially lowering the rates of false positives and false negatives.

AI algorithms, such as convolutional neural networks (CNNs), are commonly used to analyze ultrasound images by identifying and classifying lesions. The AI tool evaluated in this study, Vis-BUS, incorporates advanced algorithms for lesion detection and analysis, as well as a Cancer Probability Score (CPS) to assess the likelihood of malignancy.

2. Diagnostic Performance Metrics

In evaluating the effectiveness of diagnostic tools, several performance metrics are commonly used, including:

- Area Under the Receiver Operating Characteristic Curve (AUROC): A measure of the ability of a classifier to distinguish between classes. The AUROC value ranges from 0.5 (no better than random guessing) to 1.0 (perfect classification).
- Area Under the Precision-Recall Curve (AUPRC): Focuses on the trade-off between precision (positive predictive value) and recall (sensitivity), especially important in cases where one class is much less frequent than the other.

These metrics were used in our study to assess the diagnostic accuracy of the Vis-BUS AI tool, ensuring a comprehensive evaluation of its performance.

3. Cancer Probability Score (CPS)

CPS is a quantitative measure generated by the Vis-BUS AI tool to assess the likelihood that a breast lesion is malignant. The CPS is calculated by analyzing various features of the lesion identified in ultrasound images, such as size, shape, margin characteristics, echo patterns, and the presence of microcalcifications. The AI algorithm processes these inputs using a fully convolutional neural network (FCN) that produces a probabilistic score ranging from -100 to 100, where higher scores indicate a higher likelihood of malignancy.

3.1. Calculation Process:

- Input Data: Ultrasound images of breast lesions are processed by the AI system.
- Feature Extraction: The AI system extracts and analyzes key features of the lesions, including morphological and textural characteristics.
- Score Generation: The AI uses the extracted features to generate the CPS, which reflects the probability of the lesion being malignant. The score is derived through a combination of lesion detection (using Lesion Detection AI) and lesion analysis (using Lesion Analysis AI).
- Output: The CPS is presented on a scale from -100 to 100, aiding clinicians in assessing the malignancy risk of the lesion.

3.2. The CPS formula

$$CPS={{\theta_{LA-AI}(B}_{\mathrm{img}}, \theta}_{LD-AI}(B_{\mathrm{img}}))$$

Where, $\theta_{LA-AI}$ and $\theta_{LD-AI}$ denotes LD-AI and LA-AI neural network, respectively

4. Development of Vis-BUS

Figure 2 illustrates the overall configuration of the Vis-BUS neural network. Vis-BUS integrates two primary components: Lesion Detection AI (LD-AI) and Lesion Analysis AI (LA-AI). LD-AI uses b-mode ultrasound images, $B_{\mathrm{img}} \sim R^{256X256}$, to identify the location of the lesion, while LA-AI analyzes the breast b-mode image characteristics.

The development of Vis-BUS, an AI-driven ultrasound tool, was a multi-step process involving the integration of advanced machine learning techniques, particularly deep learning, to enhance the accuracy and efficiency of breast lesion detection and classification.

4.1. System Architecture and Components

Vis-BUS consists of two primary AI components:

- Lesion Detection AI (LD-AI): This module is responsible for detecting the location of the breast lesions in the ultrasound images.
- Lesion Analysis AI (LA-AI): This module further analyzes the detected lesions to assess their malignancy and generates the Cancer Probability Score (CPS).
  - 1. LD-AI (Lesion Detection AI):

The LD-AI module was developed based on a state-of-the-art object detection framework, leveraging deep convolutional neural networks (CNNs) to identify lesions within B-mode ultrasound images. The LD-AI component includes the following key elements:

The LD-AI is implemented based on the object detection framework [10], where the neural network outputs lesion position and size and a corresponding confidence score as an output ($L_{\mathrm{coord}})$. The LD-AI consists of a convolutional encoder backbone and a feature network. The encoder backbone is based on the EffecientNet [11] for parameter-efficient feature analysis of the $B_{\mathrm{img}}$. A bi-directional feature network [10] is employed as the feature network. The lesion location is trained to minimize mean squared error loss between the ground truth and the LD-AI output, while the confidence score employs focal loss [12] as a learning objective. The AdamW [13] with a learning rate of $1e^{-4}$ is utilized as the network optimizer.

- Convolutional Encoder Backbone: The backbone of LD-AI is based on EfficientNet, a highly efficient CNN architecture known for its balance between performance and computational cost. EfficientNet was chosen for its ability to effectively capture the complex features of ultrasound images with fewer parameters.
- Feature Network: A bi-directional feature network is employed, allowing the system to analyze features from multiple scales, improving the detection of lesions of varying sizes and shapes.
- Training Process: The LD-AI was trained using a large dataset of 19,000 annotated ultrasound images. The ground truth for lesion locations was provided by expert radiologists. The network was optimized with a learning rate set to $1e^{-4}$, focusing on minimizing the mean squared error between the predicted and actual lesion coordinates.
  - 1. LA-AI (Lesion Analysis AI):

Following the detection of a lesion, the LA-AI module processes the identified lesion to determine its malignancy, outputting a CPS. The LA-AI component includes:

The LA-AI employs $B_{\mathrm{img}}$, $L_{\mathrm{coord}}$, and lesion image to analyze the lesion malignancy. Features of each input are interpreted using a fully convolutional neural network (FCN encoder). These features are then concatenated channel-wise into a latent vector, which serves as the input for the fusion encoder, producing CPS as the neural network output. The fusion encoder architecture is based on EfficientNet. The LA-AI is optimized with AdamW optimizer (learning rate = $1e^{-3}$), with a learning objective of minimizing binary cross entropy between the CPS and the ground truth.

- Fully Convolutional Network (FCN) Encoder: The FCN processes the B-mode images and the coordinates of the detected lesions. This encoder extracts features that are crucial for malignancy assessment.
- Fusion Encoder: The output from the FCN is concatenated channel-wise into a latent vector, which is then passed through the fusion encoder. This architecture, also based on EfficientNet, integrates the features extracted from the lesion to generate a CPS.
- Optimization: The LA-AI was trained at a learning rate of $1e^{-3}$, with the objective of minimizing binary cross-entropy between the CPS and the actual malignancy status as determined by biopsy.

5. Training and Data Preparation

1. Data Sources: training dataset consisted of ultrasound images acquired from machines by leading manufacturers such as Philips (Netherlands), GE (United States), and Fujifilm (Japan). This diversity in data sources was crucial to ensure that Vis-BUS could perform reliably across different imaging systems.

2. Annotation and Ground Truth: Each ultrasound image was meticulously annotated by expert radiologists at Seoul National University Bundang Hospital (SNUBH), providing the ground truth for lesion location and malignancy. This rigorous annotation process was critical for training the AI models to achieve high accuracy in real-world clinical settings.

3. Model Validation and Testing: To ensure the robustness of Vis-BUS, the models were validated on a separate set of ultrasound images not used in training. The performance of the system was evaluated using key metrics such as the Area Under the Receiver Operating Characteristic Curve (AUROC) and the Area Under the Precision-Recall Curve (AUPRC).

1. Implementation and Integration

Vis-BUS operates by connecting to the HDMI port of conventional ultrasound imaging devices, allowing real-time processing of ultrasound video feeds. The software is installed on a tablet, which serves as the interface for clinicians. This setup enables:

- Real-Time Analysis: The AI processes the video feed in real-time, providing immediate diagnostic insights as the ultrasound examination is being conducted.
- Freeze-Frame Analysis: Clinicians can pause the ultrasound scan and perform detailed analysis on static images using the AI tool, which continues to offer diagnostic information without losing any capabilities.
- User Interface: The tablet interface is designed to be intuitive, allowing clinicians to easily navigate through diagnostic results, adjust settings, and generate detailed reports.

1. Cancer Probability Score (CPS)

The CPS, generated by the LA-AI module, quantifies the likelihood of a lesion being malignant. This score is displayed on a scale from -100 to 100, with higher scores indicating a greater probability of malignancy. The CPS is a crucial component that aids clinicians in making informed decisions about patient management.

1. Performance and Optimization

The development process included continuous iterations of training and validation to optimize the performance of Vis-BUS. The system's high AUROC and AUPRC values reflect the efficacy of the training process and the robustness of the AI algorithms in accurately diagnosing breast lesions.

For the training of LD-AI and LA-AI, 19k breast ultrasound images are acquired using ultrasound machines from Phillips (NL), GE (US), and Fujifilm (JP). The ground truth lesion location and lesion malignancy are annotated by an expert radiologist in SNUBH under IRB approval (IRB number: B-2301-807-108)

1. Explanation of Vis-BUS Operation

Vis-BUS operates by connecting a tablet, equipped with the Vis-BUS software, to the HDMI port of an existing ultrasound imaging device. This connection allows the tablet to receive the ultrasound video feed directly. Once connected, the Vis-BUS software begins to display the live video feed from the ultrasound device and can analyze the ultrasound images in real-time, providing immediate feedback and diagnostic information as the images are being captured.

Additionally, the Vis-BUS software supports analysis on freeze frames. When the ultrasound image is paused or frozen, the software can still perform detailed analysis on the static image. This feature allows for thorough examination and evaluation of specific frames without losing any diagnostic capabilities. During continuous ultrasound scanning, Vis-BUS provides ongoing analysis, highlighting areas of interest, detecting anomalies, and offering real-time diagnostic insights.

The tablet interface is designed to be intuitive and user-friendly, allowing clinicians to easily navigate through the analysis results, adjust settings, and interact with the diagnostic tools provided by the Vis-BUS software. The software can generate detailed diagnostic reports based on both real-time and frozen images, which can be saved, shared, or integrated into the patient’s medical record.

Vis-BUS also provides quantitative information on the likelihood of a lesion being benign or malignant, displaying this information on a scale from -100 to 100 on the screen. Additionally, it offers detailed BI-RADS values, assisting clinicians in making more informed diagnostic decisions.

1. Patients Dataset

The dataset for this study consisted of 258 breast ultrasound examinations Seoul National University Bundang Hospital. All data were used following Institutional Review Board approval (IRB number: B-2301-807-108), anonymized, and strictly prohibited from being shared externally. Each examination was categorized by pathologic results of biopsy as either benign (n=129) or malignant (n=129). The dataset included a diverse range of breast lesion characteristics to ensure comprehensive evaluation of the AI tool's performance across various clinical scenarios. These data were stored in the Vis-BUS analysis data folder, where each image was automatically loaded and analyzed.

**Supplementary Material S3. Statistical Analysis**

This section provides a detailed overview of the statistical methods employed to assess radiologists’ diagnostic performance with and without AI assistance in our multi-reader, multi-case (MRMC) study. We analyzed the data using MRMC analysis of variance techniques for primary ROC comparisons, and additional models for subgroup analyses, pooled accuracy metrics, inter-reader reliability, reading time, and localization performance.

**MRMC Study Design and Primary Analysis (AUROC)**

We conducted a fully-crossed MRMC reader study in which six radiologists each read the same set of 258 ultrasound cases under two conditions (unaided and AI-assisted). In this design, every reader interprets every case in both modalities, resulting in cross-correlated data in which readers and cases are considered random effects . An MRMC analysis accounts for variability and correlation from both readers and cases when estimating performance differences, by treating these factors as random and adjusting variance estimates accordingly .

The primary performance metric was the area under the receiver operating characteristic curve (AUROC) for distinguishing malignant vs. benign lesions. We computed reader-specific AUROC values for each condition (with and without AI) using empirical ROC analysis of the continuous malignancy scores. To compare AUROC across the two reading conditions, we utilized the Obuchowski–Rockette (OR) MRMC method . The OR method is an ANOVA approach for multi-reader diagnostic accuracy data that accounts for the non-independence of readings; it treats readers and cases as random effects and provides a unified analysis of variance framework for comparing modalities . In our implementation (using the MRMCaov R package, version 0.3.0), we obtained the mean AUROC for each modality (averaged across readers) and used OR analysis to test the hypothesis of no difference in AUROC between unaided and AI-assisted readings . The OR analysis yielded an F-statistic for the modality effect and was used to compute two-sided p-values and 95% confidence intervals for the difference in reader-averaged AUROCs. This approach, originally proposed by Obuchowski and Rockette (1995) and later expanded by Hillis et al., is well-suited for MRMC ROC comparisons and has been widely adopted in radiology observer studies . We report the estimated difference in AUROC (∆AUROC) between conditions along with its confidence interval and p-value from the OR analysis as the primary outcome.

**Subgroup Analyses**

We performed predefined subgroup analyses to explore AI performance within specific case categories. Cases were stratified by factors such as patient age, lesion size, tumor stage, breast density, and baseline BI-RADS assessment. For example, we compared performance on small tumors versus larger tumors (using T1 ≤ 2 cm vs. >2 cm categories), on cases in dense breast tissue (BI-RADS density C/D) versus nondense (A/B), and across patient age groups (e.g., ≤50 vs. >50 years). Within each subgroup, we recalculated key diagnostic metrics for radiologists with and without AI. AUROCs were computed per subgroup and compared between modalities using the same OR MRMC approach applied to the subset of cases. For binary accuracy measures like sensitivity (for cancer cases) and specificity (for benign cases), we used methods analogous to the overall analysis but confined to the subgroup. In particular, we applied logistic regression with generalized estimating equations (GEE) to compare sensitivities between unaided and AI-assisted reads in subgroups . For instance, in the subgroup of small (T1) cancers, readers’ sensitivity with vs. without AI was compared using a GEE model adjusting for multiple readers . These subgroup analyses were considered exploratory; no adjustments were made for multiple comparisons, so the results are interpreted with caution.

**Pooled Sensitivity, Specificity, PPV, and NPV (GEE Analysis)**

Beyond reader-averaged AUROC, we estimated pooled diagnostic performance metrics (sensitivity, specificity, positive predictive value [PPV], and negative predictive value [NPV]) across all readers for each condition. To do so while accounting for the MRMC data structure, we employed population-averaged GEE models . Each individual case interpretation (a reader’s diagnosis on a case) was treated as an observation with a binary outcome (e.g., “correctly classified as malignant” for sensitivity among cancer cases, or “correctly classified as benign” for specificity among non-cancer cases). We specified an exchangeable correlation structure and clustered by case to account for the fact that multiple readers evaluated the same cases. This approach produces pooled estimates that reflect the average performance across readers, with robust standard errors accounting for inter-reader correlation . We fit separate logistic GEE models for each metric: for sensitivity, considering only malignant cases (outcome = 1 if the reader identified the cancer, 0 if missed); for specificity, considering only benign cases (outcome = 1 if correctly identified as benign, 0 if falsely marked malignant); and similarly for PPV and NPV (where the denominators are positive and negative interpretations, respectively). From each model, we obtained the estimated proportion (e.g., sensitivity) with 95% confidence interval. For example, pooled sensitivity across all readers in a given condition was calculated using a GEE model and reported with its 95% CI . To compare these proportions between the unaided and AI-assisted modalities, we included the reading modality as a covariate in the GEE and assessed its coefficient (which represents the log odds ratio for detection with AI vs. without). In practice, this yielded p-values for differences in sensitivity or specificity between conditions, adjusted for the clustered nature of the data. We implemented the GEE analysis using the **geepack** package (version 1.3-2) in R, and cross-verified results with the **DTComPair** package for paired diagnostic accuracy data .

**Classification Thresholds and Binary Outcomes**

Radiologists provided continuous probability of malignancy scores (either explicitly as the AI’s Cancer Probability Score or implicitly via BI-RADS/Likert ratings), which we dichotomized to evaluate binary classification performance. We defined a *positive* test as one that would lead to a biopsy recommendation. For the AI’s Cancer Probability Score (CPS), which ranges from –100 to 100, we used a threshold of 0: any case with CPS > 0 was considered test-positive (malignant), whereas CPS ≤ 0 was considered test-negative (benign) . This threshold corresponds to the AI’s decision boundary, effectively classifying cases with an estimated >50% malignancy likelihood as positive. For radiologists’ interpretations, we considered an assessment positive if the reader indicated suspicion warranting biopsy (e.g., BI-RADS category 4 or 5, or in our 7-point Likert scale, a score ≥4 for malignancy). Using these thresholds, each case read was labeled as either malignant or benign prediction, which enabled calculation of sensitivity, specificity, PPV, NPV, and overall accuracy based on 2×2 contingency tables . All binary classification metrics were computed per reader per modality, and then pooled or compared using the methods described above.

**Reading Time Analysis**

We evaluated whether AI assistance led to a significant change in interpretation time per case. Each reader recorded the time taken to interpret each case in both sessions. Because the same readers interpreted the same cases with and without AI (after a washout period), we treated the reading times as paired observations. The distribution of reading times was right-skewed, so we opted for non-parametric analysis. We first calculated, for each case-reader pair, the difference in reading time between the unaided and AI-assisted readings. We then applied the Wilcoxon signed-rank test to these paired differences to test if the median difference in reading time was significantly different from zero . This test accounts for the paired nature of the data and is appropriate for non-normal distributions. We supplemented the hypothesis test with an estimate of the median time savings due to AI and its confidence interval. Specifically, we used bootstrapping to derive a 95% confidence interval for the median difference in reading time. We performed 10,000 bootstrap resamples (sampling pairs of observations with replacement) and computed the median difference for each resample. The 2.5th and 97.5th percentiles of the bootstrap distribution of median differences were taken as the confidence limits. This approach (implemented with the boot package, version 1.3-31) makes minimal assumptions about the underlying distribution of times. We report the median reduction in reading time per case with AI (if positive, indicating faster readings with AI) along with the bootstrapped 95% CI and the p-value from the Wilcoxon test for significance of the paired difference.

**Software and Statistical Tools**

All statistical analyses were conducted using R (R Foundation for Statistical Computing, Vienna, Austria). We leveraged several specialized packages and tools for MRMC and diagnostic performance analysis. The primary ROC analysis was performed using the MRMCaov package (version 0.3.0) for multi-reader ROC ANOVA . The iMRMC software (version 2.1.0) was also used in parallel to verify MRMC AUROC results and variance estimates ; iMRMC provides a Java-based implementation of MRMC analysis consistent with methods by Obuchowski, Rockette, and Hillis. For logistic GEE models of sensitivity and specificity, we used the geepack package (version 1.3-2) and DTComPair (version 0.1.3) in R . The irr package (version 0.84.1) was used to compute ICC for inter-rater reliability, and the boot package (version 1.3-31) was used for bootstrap confidence intervals. All statistical tests were two-tailed, with a significance level of α = 0.05. Key statistical parameters, definitions, and formulas are summarized in Table 1 for reference.

**Table S2. Key statistical metrics and their definitions/formulas.**

| **Parameter** | **Definition / Formula** |
| --- | --- |
| **Area Under the ROC Curve (AUROC)** | The area under the receiver operating characteristic curve, summarizing the trade-off between sensitivity and 1–specificity across all possible classification thresholds. It can be interpreted as the probability that a randomly selected positive case is rated higher than a randomly selected negative case. |
| **Sensitivity** (True Positive Rate) | Proportion of actual positives (malignant cases) correctly identified as positive. *Formula:* $\displaystyle \frac{\text{True Positives}}{\text{True Positives} + \text{False Negatives}}$. |
| **Specificity** (True Negative Rate) | Proportion of actual negatives (benign cases) correctly identified as negative. *Formula:* $\displaystyle \frac{\text{True Negatives}}{\text{True Negatives} + \text{False Positives}}$. |
| **Positive Predictive Value (PPV)** | Probability that a case is truly malignant given that it was classified as positive. *Formula:* $\displaystyle \frac{\text{True Positives}}{\text{True Positives} + \text{False Positives}}$. |
| **Negative Predictive Value (NPV)** | Probability that a case is truly benign given that it was classified as negative. *Formula:* $\displaystyle \frac{\text{True Negatives}}{\text{True Negatives} + \text{False Negatives}}$. |
| **Wilcoxon Signed-Rank Test** | A nonparametric test for paired data that assesses whether the median of the differences between paired observations is zero. Used here to test for median reading time differences between conditions. |

**Supplementary Material S4: Calibration analysis**

Calibration analysis showed that the AI tended to overestimate malignancy probabilities at higher CPS values (Supplementary Fig. S2). When using a CPS threshold of +20 to recommend biopsy, sensitivity and specificity were 91.5 % and 68.2 %, respectively, which approximates BI RADS 4A recommendations. Further work is needed to refine threshold settings.

**Supplementary Fig. S1**
